# Mobilizing registry data for quality improvement: A convergent mixed-methods analysis and application to spinal cord injury

**DOI:** 10.3389/fresc.2023.899630

**Published:** 2023-04-03

**Authors:** Jacqueline A. Krysa, Kiran J. Pohar Manhas, Adalberto Loyola-Sanchez, Steve Casha, Katharina Kovacs Burns, Rebecca Charbonneau, Chester Ho, Elizabeth Papathanassoglou

**Affiliations:** ^1^ Neurosciences, Rehabilitation and Vision, Strategic Clinical Network, Alberta Health Services, Edmonton, AB, Canada; ^2^ Division of Physical Medicine and Rehabilitation, University of Alberta, Edmonton, AB, Canada; ^3^ Community Health Sciences, Cumming School of Medicine, University of Calgary, Calgary, AB, Canada; ^4^ Department of Clinical Neurosciences, Hotchkiss Brain Institute, Cumming School of Medicine, University of Calgary, Calgary, AB, Canada; ^5^ School of Public Health, University of Alberta, Edmonton, AB, Canada; ^6^ Department of Clinical Quality Metrics, Alberta Health Services, Edmonton, AB, Canada; ^7^ Faculty of Nursing, University of Alberta, Edmonton, AB, Canada

**Keywords:** quality improvement, patient registry data, interdisiciplinary teams, spinal cord injury (SCI), complex chronic conditions

## Abstract

**Introduction:**

The rising prevalence of complex chronic conditions and growing intricacies of healthcare systems emphasizes the need for interdisciplinary partnerships to advance coordination and quality of rehabilitation care. Registry databases are increasingly used for clinical monitoring and quality improvement (QI) of health system change. Currently, it is unclear how interdisciplinary partnerships can best mobilize registry data to support QI across care settings for complex chronic conditions.

**Purpose:**

We employed spinal cord injury (SCI) as a case study of a highly disruptive and debilitating complex chronic condition, with existing registry data that is underutilized for QI. We aimed to compare and converge evidence from previous reports and multi-disciplinary experts in order to outline the major elements of a strategy to effectively mobilize registry data for QI of care for complex chronic conditions.

**Methods:**

This study used a convergent parallel-database variant mixed design, whereby findings from a systematic review and a qualitative exploration were analyzed independently and then simultaneously. The scoping review used a three-stage process to review 282 records, which resulted in 28 articles reviewed for analysis. Concurrent interviews were conducted with multidisciplinary-stakeholders, including leadership from condition-specific national registries, members of national SCI communities, leadership from SCI community organizations, and a person with lived experience of SCI. Descriptive analysis was used for the scoping review and qualitative description for stakeholder interviews.

**Results:**

There were 28 articles included in the scoping review and 11 multidisciplinary-stakeholders in the semi-structured interviews. The integration of the results allowed the identification of three key learnings to enhance the successful design and use of registry data to inform the planning and development of a QI initiative: enhance utility and reliability of registry data; form a steering committee lead by clinical champions; and design effective, feasible, and sustainable QI initiatives.

**Conclusion:**

This study highlights the importance of interdisciplinary partnerships to support QI of care for persons with complex conditions. It provides practical strategies to determine mutual priorities that promote implementation and sustained use of registry data to inform QI. Learnings from this work could enhance interdisciplinary collaboration to support QI of care for rehabilitation for persons with complex chronic conditions.

## Introduction

Complex chronic conditions are a leading cause of morbidity and mortality worldwide (1, 2). In Canada, 13% of individuals report living with two or more chronic conditions (3). These conditions influence diverse facets of an individual's experience and ability to participate in daily life, while also disrupting the delivery of usual care and decision making (4, 5). Spinal cord injury (SCI) is a highly disruptive and debilitating complex chronic condition that directly impacts lifelong physical, psychological, and social well-being (6, 7). SCI and other complex chronic conditions require significant rehabilitation to address functional and psychosocial goals that enhance participation in the community and daily life (8). Rehabilitation care for complex conditions spans across diverse settings including inpatient, outpatient, and the community (9). Primary care is integral to supporting the coordination of care between patients, rehabilitation care providers, and community care partners in the management and rehabilitation of patients with SCI (10). Enhancing the quality and integration of care services to support complex chronic conditions is essential to achieving health system change and improving patient outcomes (11, 12). Notable barriers to integration of care include lack of organizational support, limited resources, scarcity of theoretical frameworks for evaluating existing data, data and privacy restrictions, and inadequate or siloed informational technology structures to support continuous health information sharing (13). These barriers require significant investment from leading health care systems to enhance quality of care services and patient outcomes (14, 15).

Significant change in health system quality of care through decision-making and quality improvement (QI) initiatives must be informed by robust, and reliable evidence (16). Learning health systems support infrastructures that foster data sharing, and knowledge creation to enhance evidence-informed decision making to improve health outcomes (17) and promote quality of care (18). Considering patient populations with chronic, life-long health conditions, a learning health system is critical for service improvement of the care that is often lacking for persons with complex chronic conditions (19). While several jurisdictions are beginning to adopt the learning health system paradigm and capture needed data, a predictor of success of such approaches is the speed at which learning occurs and appropriate adjustments are made (20). The diversity of rehabilitation care for complex conditions warrants the continued measurement of the patient's functional performance and health outcomes across the care continuum (21). Health administrative data describes patient information that is normally collected by government and health care providers to inform provider payment and the management of patient care (e.g., physician billing claims, hospital discharge records) (22). Generally, administrative health data does not capture the wide array of measures to address these complex research queries and warrants additional data sources to support continuous QI of care (23), such as specialized patient registries.

Patient registries, or clinical data registries, are organized systems or interactive databases that use observational study methods to systematically collect and monitor clinical data (e.g., medical history, patient-reported data, laboratory values) of a population that is usually defined by a particular condition or exposure (24). The purpose of clinical data registries is to monitor, evaluate, and improve outcomes of a specific population over time (25). Registry databases can be developed from electronic health records to support various purposes such as public health, health services research, health promotion, patient care, clinical research, and public safety (24).

Registry databases are increasingly being used for clinical monitoring and QI. Information from registry databases can be used to develop and inform QI strategies that improve clinical outcomes and reduce variation in care. Despite the existence of several registries on complex, chronic conditions, there is limited literature explicitly describing their direct application for QI initiatives (26–28). The use of patient registries for health system decision making is complex, and a growing barrier is widespread awareness and application of this data to inform health system QI of care (26). Although the use of registry databases has been found to positively impact care, there are continued challenges for oversight of its use for complex conditions, such as SCI, requiring coordination of care amongst primary care, community care, and other specialist health providers (29). A notable SCI registry, the Rick Hansen Spinal Cord Injury Registry (RHSCIR), is Canada's first nation-wide registry and has been capturing data on patients with SCI since 2004 (30). Despite the robust and unique patient data captured within RHSCIR, there remain challenges on how to consistently use and implement this data for evidence-informed decision making and QI (31). The aim of this study is to describe approaches to mobilizing registry data with interdisciplinary partners to inform QI of rehabilitation care for persons with complex, chronic conditions, using SCI as a case study. These learnings will provide insights into the barriers and facilitators for using registry data for QI to support continuous data sharing with interdisciplinary partners for QI of SCI quality of care.

## Methods

### Study design

This study uses a convergent mixed-methods design using a parallel-database variant, whereby findings from a scoping review and qualitative description are analysed independently, and then results of the complimentary databases are compared (32, 33). This allowed for a better understanding of the research question that would be less apparent from analysis of each dataset al.one. This study was approved by the University of Alberta Research Ethics Board (Pro00107626). All participants were informed of the study and provided written consent.

### Setting and organization

This study was conducted through Alberta Health Services (AHS) in Alberta, Canada. AHS is Canada's first and largest province-wide integrated health care system and currently provides care services to over 4 million Albertans (34). In Canada, rehabilitation care for persons with complex conditions is delivered across diverse care settings including in-hospital, outpatient clinics, primary care, and specialized community care programs (8).

### Scoping review

#### Data sources and search strategy

**Figure 1:**
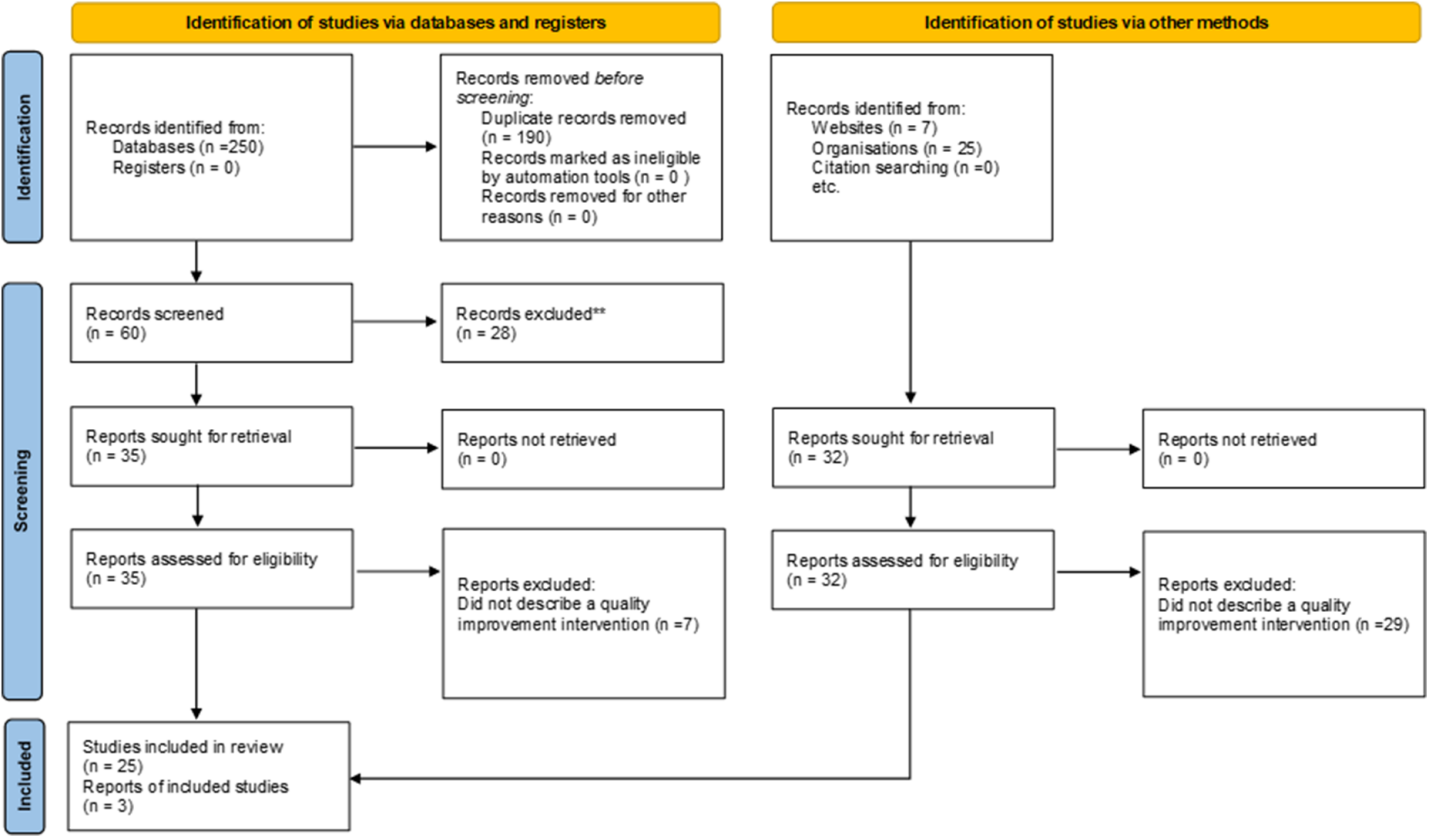
PRISMA flow chart [adapted from Page et al. (35)].

A systematic scoping review was performed according to JBI methodology (Figure 1) (36). A health librarian developed and conducted the literature search for academic and grey literature. Peer-reviewed publications were searched within four databases: MEDLINE (Ovid); PubMed; CINAHL; MEDLINE (Ebsco). The grey literature was reviewed by searching various government, health organization, QI, and SCI registries (see Supplementary Appendix A for a complete list for Grey Literature sources). Search terms to capture articles about using registry data for QI for SCI and other complex chronic conditions included “registry data”, “quality improvement”, “continuous improvement”, “implementation”, “chronic condition”, and others. Please see Supplementary Appendix B for a full list of search terms.

#### Study selection

Inclusion and exclusion decisions were recorded using a standard coding system based on PICO (population/patients, intervention, comparison, and outcomes) criteria (37). The population included individuals (adults and pediatrics) with SCI or complex, chronic conditions. The intervention was specific to QI or related approaches. The presence or absence of a comparator did not determine eligibility. The outcome of interest was the strategy, approach, implementation and/or outcome of the QI approach. Articles that discussed registry data elements but did not directly link the registry items to a QI process or outcome were excluded. Databases were searched from January 2010 to January 2021 to ensure the most up-to-date strategies were reviewed. Inclusion criteria included articles published in English, from North America (Canada, United States), Europe, Japan, Australia, and New Zealand. Articles were limited to original research, reviews, or reports. Conference proceedings, theses, and news articles were excluded. A three-staged process was used to screen and extract articles. Due to feasibility, one reviewer (JAK) conducted all stages, and results were confirmed with the team. Title and abstracts were screened for relevance to advance to full-text review. Included articles underwent data extraction. All search results were stored and managed within Mendeley and Microsoft Excel. Data extraction was performed using an *a priori* data extraction form including: publication date; country; development organization and process; target population; QI intervention; and outcome measurement(s). Articles were screened for outcomes of using registry data for QI, perceived barriers and facilitators to using registry data for QI, and approaches of QI implementation.

### Qualitative interviews

Semi-structured interviews (38) were conducted to clarify stakeholder perspectives in designing and implementing QI initiatives that leverage patient registry data at the organizational or provincial level.

#### Sampling and recruitment

Purposive sampling initially and a subsequent snowball approach were used to capture the diverse experiences of multidisciplinary stakeholders with mobilizing registry data for QI as well as those interested in supporting this process. A snowball sampling approach was further employed to enhance the sampling during the interview process. Participants with interest or experience using patient registry data for any complex chronic conditions were eligible to participate. There were no explicit exclusion criteria. Relevant professional stakeholders were identified through the scoping review as well as through a broader team of clinicians, researchers, and decision makers in SCI care in Alberta, Canada. Targeted roles included directors of condition-specific national registries, senior QI researchers, clinical champions, members of the national SCI community, leadership from SCI community organizations, and persons with lived experience of SCI. During the interview process, interviewees were asked to identify and connect the research leads to additional stakeholders. A community SCI organization (SCI Alberta) based in Alberta, Canada advertised and facilitated recruitment of persons with lived experience of SCI. The research team received the contact information of participants who had consented to being contacted, and thereafter initiated email discussions for written consent and interview coordination.

#### Data collection

Interviews took place between April and June 2021. A semi-structured interview guide with open-ended questions was developed based on relevant literature (39–41) as well as discussions among subject-matter experts (national registry directors, provincial QI consultants, researchers, clinicians, SCI community organizations, and persons with lived experience of SCI). The interview guide covered several topics, including: knowledge and experiences of using registry data for QI; approaches for prioritization; barriers and facilitators to using registry data; implementation strategies; and effective strategies to promote motivation and on-going collaboration to support QI (see Supplementary Appendix C for complete interview guide). One-on-one interviews were conducted over virtual platforms (Zoom) by an experienced interviewer trained in qualitative methods (JAK). At their request, two participants from the same organization participated together in a single interview. Interviews were 30–90 min long. All interviews were audio-recorded, and confidentially transcribed verbatim. Participant names and identifiers were removed from the transcripts prior to analysis. Recruitment and interviews continued until thematic saturation, wherein no new codes or themes emerged from the interviews (42).

#### Data analysis

Stakeholder interviews were transcribed, categorized by theme, and compared between different interviewees and professional roles using a qualitative description methodology to ensure validity and credibility (43). Credibility was achieved by using a semi-structured interview guide with open-ended questions to facilitate prolonged engagement with the participants. To satisfy the criterion of transferability in-depth description of data and presenting exemplar quotes for identified themes. Dependability was satisfied by independently coding two transcripts by three manuscript authors (JAK, EP, KPM) into meaning-bearing units (codes) related to the study objectives. These codes were compared and discussed until agreement on relevance and appropriateness was achieved by all researchers. This subset analysis built a set of defined codes that were applied to subsequent interviews analyzed by one researcher (JAK) and developed into major themes. Connections within and in-between themes were examined to explore relationships or causality to gain an in-depth understanding of stakeholders experience and perspectives on using registry data for QI. Qualitative analysis was facilitated using NVIVO-12 software (QSR International).

### Convergent data analysis

Results from the scoping review and qualitative interviews were analyzed separately and then simultaneously for synthesis and interpretation, which allowed for comparative analysis of individual datasets for discussion (32, 33). This comparative analysis enabled the integration of results to create a clearer understanding of how registry data is used for QI for complex conditions amongst interdisciplinary care teams. Findings from the scoping review and qualitative interviews are reported in the results section and the convergent analysis and implications are presented in the synthesis and discussion section (32).

## Results

### Scoping review

A total of 282 records were reviewed, including 250 from the academic literature and 32 from the grey literature, 190 duplicates were removed. After screening, 35 articles and 29 reports were excluded due to wrong intervention (i.e., the study was not using registry data to inform the planning or development of a QI initiative). The remaining 25 articles and 3 reports underwent full-text extraction (Figure 1, and Supplementary Appendix D). The median year of publication was 2017 (range: 2010–2020). Most publications originated from the USA (*n* = 9), Canada (*n* = 5) or Sweden (*n* = 5). Of the 28 studies reviewed, 11 described QI initiatives that involved registry data (Table 1). Other studies included qualitative analysis of barriers and facilitators to using registry data for QI (*n* = 7); discussions of how registry data has been used to improve care processes and clinical outcomes (*n* = 8); as well as how registry data was prioritized to inform QI initiatives (*n* = 2) (see Supplementary Appendix D for a full list of reviewed studies).

**Table 1:** Quality improvement approaches, level, timeline, aims, and use of registry data.

Author	Registry Name	Registry Condition(s)	Level of Intervention	Time	Aim(s)	Use of Registry Data
Algurén et al., 2019 (44)	Swedish Heart Failure Registry Better Management of Patients with OsteoArthritis Registry	Heart Failure Osteoarthritis	Health System	6 months 1 year	To compare two quality improvement collaboratives using National Quality Registries	Monitor and evaluate program outcomes
Australian OrthopaedicAssociation (2020) (45)	National Joint Replacement Registry	Arthroplasty	Health System	1 year	To assess the feasibility of establishing national data collection for patients having joint replacement surgery	Monitor and evaluate clinical performance of patient reported outcome measures
Burry et al., 2015 (28)	Systematic review of pediatric diabetes registries	Pediatric Diabetes	Patient, Provider, Health System	Varied	To evaluate the impact of quality improvement initiatives that use data from pediatric diabetes registries on health care processes, organization of care, and patient outcomes	Monitoring and evaluating clinical and implementation outcomes
Dykes et al., 2018 (46)	American College of Radiology National Radiology Data Registry	Radiology Patients	Health System	1 year	To develop 3 national performance benchmarks of intravenous, iodinated contrast extravasation during CT exams	Used to monitor and evaluate clinical outcomes
Etz et al., 2015 (47) (protocol)	Protocol to develop a Type 2 diabetes registry	Type 2 Diabetes	Health System	5 years (expected)	To evaluate the effectiveness and sustainability of a quality improvement initiative to support work process change in primary care practice and enhance population-based care by implementing data from a diabetes registry	Used to monitor and evaluate patient outcomes
Fonarow et al., 2010 (48)	Improve the use of evidence-based heart failure therapies in the outpatient setting	Heart Failure	Health System	2 years	To achieve an improvement of 20% in at least 2 of 7 quality measures	Used to monitor and evaluate clinical outcomes
Haggstrom et al., 2010 (49)	16 community health centers created a practice registry using software provided by the Health Disparities Cancer Collaborative	Cancer	Health System	1 year	To understand key measurement issues of stakeholders participating in quality improvement interventions	Monitor and evaluate clinical outcomes
Kaplan et al., 2018 (50)	Ohio Birth Registry	Birth Registry Data	Health System	1 year	To evaluate the success of a quality improvement initiative at reducing early elective deliveries and improve birth registry data and accuracy	Monitor and evaluate clinical outcomes
Martin et al., 2019 (51)	Improving pediatric and adult congenital treatments	Congenital Heart Disease	Health System	6 years	To reduce mortality and improve quality of life of infants with hypoplastic left heart syndrome	Inform sites of ongoing surgical care processes
Meerhoff et al., 2017	Developed a registry for physical therapists	Physical therapy patient reported outcome measures	Provider	4 years	To describe the development of an implementation strategy for the program to evaluate the feasibility of building a registry and implementing patient reported outcomes in physical therapist practice	To monitor and evaluate performance
Noonan et al., 2012 (52)	Rick Hansen Spinal Cord Injury Registry	SCI	Health System	Unclear	To develop a health care delivery model for the SCI continuum of care	Used to develop process maps to describe patient flow, resource availability and utilization of services; validated the ACT model

#### Uses of registry data for quality improvement

Table 1 describes the uses of registry data on patients with complex conditions for QI of care. A common objective of using registry data was to improve clinical outcomes such as quality of life, patient mortality, and other specific clinical outcomes for persons living with complex chronic disease (28, 44, 47, 50, 51, 53–54). Registry data was used to monitor and improve the organization and processes of clinical care including reducing regional and site variation of care provision; access to care; implementing and monitoring best practice standards; optimizing healthcare utilization and; reducing healthcare related costs (28, 31, 49, 51, 53, 55, 56). Registry data was used to develop and monitor performance indicators to evaluate care processes and inform clinical and health policy decision making (28, 45, 46, 48, 56, 57–60). This included describing the development and testing of performance indicators or suitable platforms to use performance indicators (45, 56, 60). Performance indicators were developed to assess adherence to recommended practice guidelines (53, 58), improve performance in clinical procedures (46), improve clinical outcomes (48), or assess variation in care between sites (59). Registry data was also used to develop and monitor QI implementation strategies (31, 46, 47, 52).

#### Approaches to using registry data for QI

Several articles described or evaluated a single QI initiative (*n* = 5); five reviewed a collection of QI initiatives using registry data; and one described a protocol for an ongoing QI initiative. Table 1 describes these 11 QI approaches. Most QI implementation strategies reviewed (*n* = 9) targeted change at the health system level using processes such as audit and feedback, learning seminars, training, and educational materials. Few studies (*n* = 1 study; *n* = 1 review) described QI interventions targeted at the provider (28, 60), or patient level (*n* = 1 review) (28). There were six studies that discussed collaboration with national registries (28, 44, 46, 48, 51, 52), as well as one state-wide registry (50). In two studies, a third-party agency developed registry data software (49, 60). Sites participating in the QI intervention used this software to input, monitor, and evaluate patient registry data to allow for multi-site comparison (49, 60). There was one published study protocol that detailed a methodology to support the development and management of registry data collection using internal health system resources to support long-term sustainability (47). Most interventions were one year in length, although one described an implementation process up to six years long (51). One protocol discussed how initial adoption of a registry at a clinical site can take up to 3 months (47).

This review revealed several types of QI initiatives including QI collaboratives (44), continuous QI (28), practice QI (46), and Learning Collaboratives to support care of persons with complex chronic conditions (Table 2) (53). Four studies implemented QI initiatives along with Plan-Do-Study-Act (PDSA) cycles (28, 44, 49, 50). Several studies were informed by QI frameworks including the Institute for Healthcare Improvement's (IHI) Breakthrough Collaborative Model (49, 51), the Knowledge-to-Action Framework (52), and the Grol and Wensing Implementation of Change Model (60). Detailed descriptions of these QI methodologies are discussed further in Supplementary Appendix E. The most common implementation strategy mentioned to advance QI initiatives was audit and feedback (*n* = 8) (28, 44–46, 48–50, 60). Audit and feedback was used to share and evaluate clinical site performance over time and provided an opportunity for participating sites to develop targeted strategies to improve processes over time. Audit and feedback data was often provided as a data report, which were shared with participating sites (in person or virtual) throughout the implementation period (45, 48, 53). These reports would highlight data trends of interest and in some cases, compare registry data with source documentation to determine compliance with best practice (48). One study used registry data to develop process maps to describe patient flow, resources, and utilization of services across provincial care sites (52). Additional implementation strategies included mentorship, training and educational materials, meetings and workshops, and others listed in Table 2.

**Table 2:** QI processes informed by registry data.

Author	QI Approach	Implementation Activities
Algurén et al., 2019 (44)	QI Collaborative, Plan-do-study-act (PDSA) cycles	Leadership from QI experts, on-site training, emails, audit and feedback, learning seminars
Australian Orthopedic Association (2020) (45)	Not explicitly stated	Steering committee, working group, instrument subgroup, on-site training, audit and feedback, dashboard development, survey
Burry et al., 2015 (28)	PDSA cycles, Continuous QI	Audit and feedback, education, financial incentives, self-management promotion, learning sessions
Dykes et a., 2018 (46)	Practice QI	Audit and feedback, educational materials
Etz et al., 2015 (47)	Support practices to adopt registry-based care intervention	On-site training, peer mentors, self-assessment checklists, meetings
Fonarow et al., 2010 (48)	Practice improvement interventions (*via* IMPROVE-HF toolkit)	Clinical decision support tool kit, educational materials, audit and feedback, educational and collaborative opportunities
Haggstrom et al., 2010 (49)	IHI's Breakthrough Collaborative Model (three national, in-person sessions to implement practice-level interventions) (61), PDSA Cycles	In-person meetings and workshops, audit and feedback
Kaplan et al., 2018 (50)	IHI's Breakthrough Collaborative Model (improvement strategies tested at individual sites were shared with other participating sites), PDSA Cycles	One-on-one coaching, face to face meetings, regular group webinars, audit and feedback, QI consultant
Martin et al., 2019 (51)	IHI's Breakthrough Collaborative Model (semiannual face-to-face meetings and monthly conference calls, monthly team reports, assessments) (61)	Task force, patient and family advisor engagement, face to face meetings, conference calls, regular team reports, assessments
Meerhoff et al., 2017	Grol and Wensing implementation of change model (62)	Educational workshops, peer assessment audit and feedback, knowledge brokers, workshops, self-assessment questionnaires
Noonan et al., 2012 (52)	Knowledge to Action Process	Level setting, process mapping, validation of model by site personnel, educational toolkits

### Qualitative interviews

The present study included *n* = 11 participants that varied in profession, organizational affiliation, and geographic location. Characteristics of study participants are reported in Table 3. Participants were from Canada (*n* = 8), United States (*n* = 2), and Germany (*n* = 1). Most participants were professional stakeholders with experience relevant to coordinating SCI care in the community (*n* = 6), leading or supporting QI initiatives using SCI patient registries at the institutional (*n* = 1), provincial (*n* = 2), and federal level (*n* = 2). An individual with lived experience of SCI (*n* = 1) was also interviewed. The mean years of professional experience was 14.3 years (11.3 SD). Interviews ranged from 30 to 90 min long.

**Table 3:** Characteristics of participants.

*N*	11
Gender (% Female)	82%
Professional Experience (Years [mean (SD)]	14.3 (11.1) years; range: 2–37 years
Professional Affiliation	Community Organization (*n* = 5) Federal Government (*n* = 2) Provincial Research Institute (*n* = 2) University/Academic Research Center (*n* = 1) Lived Experience of SCI (*n* = 1)
Geographic Location	Canada (*n* = 8) USA (*n* = 2) Germany (*n* = 1)

The identified themes highlight key findings from the stakeholder perspectives regarding effectively mobilizing registry data for QI of SCI care. Three key themes were identified: registry data access and validity; communication and collaboration; and operationalization and sustainability (Please see Supplementary Appendix F for additional exemplar quotes to support these themes).

### Theme 1: registry data access and validity

When discussing applications of registry data for QI, participants generally experienced difficulty accessing registry data and often described registry data as having limited data quality, validity and reliability for use for organizational or higher-level QI.

#### Limited high-quality evidence

The current state of SCI evidence was described as a barrier to using SCI registry data for QI. Many stakeholders attributed the gaps to developing QI initiatives for SCI care to a perceived lack of high-quality SCI data available to inform clinical practice guidelines and appropriate benchmarks of care. This included data from low-quality studies, studies with limited sample size, or a lack of comparator groups. Participants also voiced concerns on the limited data available on persons with non-traumatic SCI. Participants described challenges identifying and monitoring patients with non-traumatic SCI as this demographic is often treated outside the traditional SCI care trajectory and therefore, are often excluded or have minimal representation in SCI patient registries. This has resulted in sparse patient information to inform clinical outcomes and subsequently limited evidence to inform the development of care standards for persons with non-traumatic SCI.

*“SCI is an area that struggles from having limited research to draw from… [it is] is either poor quality research… [has] very small sample sizes, [or] no comparison groups…”* (Participant #5, Federal Government Researcher)

#### Registry data access and reliability

Another common barrier to using registry data for QI was access to registry databases as well as the reliability of the registry data. Most participants were frustrated by the bureaucratic barriers to access and use registry data as well as the cost to use and implement the data. There was also discontent about the internal processes to update and change variables to align with clinical practice change.

 “*Different [registry] data sets are stewarded by different organizations and [can take] years to get…full approval to access [and] link these data sets.*” (Participant #3, National SCI Registry Senior Leadership)

Registry data quality was identified as a significant barrier for QI implementation. Missing or incomplete data for priority variables of SCI care was a barrier to registry data use for QI. Additional frustrations centred on the time required for registry data cleaning, validation, and data management.

“*We would pull from the old registry data and things like the ASIA score would be missing 60% of the time…so a lot of times we wouldn’t report it.*” (Participant #5, Federal Government Researcher)

#### Data validation

To enhance data reliability and validity, participants suggested to validate registry data with the administrative data sources to compare patient volumes and determine whether the registry data source is reflective of the clinical population at sites participating in the QI initiative.

“*It's really important…to validate the data that you found and after [that], validate the interpretation and the analysis of the data with administrative data sources.”* (Participant #8, Provincial Research Institute Researcher)

### Theme 2: communication and collaboration

Ongoing communication and collaboration were identified as a major theme to support the implementation of novel QI initiatives. Broader registry data use was described to be limited by provider motivation and competing priorities to use registry data as well as issues with implementation feasibility. Conversely, leveraging interdisciplinary collaboration; champions for accountability and sustainability; mutual priorities and incentives; as well as frequent and ongoing collaboration were described to support the planning and development of QI initiatives.

#### Provider motivation and competing priorities

Many participants felt provider motivation (or lack thereof) significantly impeded use of registry data for QI initiatives. Participants believed that this was due to limited provider confidence in the capacity of registry data being used to inform practice change. Participants described their own hesitancy to add additional responsibilities to providers with already-full workloads as well as avoiding duplication of existing work. These barriers limited overall provider motivation to participate in QI-related activities that leverage registry data.

*“Think about the minimal data that is needed in order to answer this question. Don’t make the mistake and acquire a lot of data where you might think that you will need it at some point in time because then people are really reluctant and overloaded to collect these data.” (Participant #10, National SCI Registry Lead)*

#### Mutual priorities and incentives

QI prioritization was determined through both top-down and bottom-up approaches and included input from stakeholders, clinicians, and patients. Many participants emphasized engaging with patients throughout the QI development process.

“[It is] *important to understand the key elements in the [care] trajectory for both the experts and the patients… What's important for the patient is not the same as what's important for the physician.*” (Participant #11, Federal Government Researcher)

Participants conducted engagement activities with stakeholders, such as interviews or focus groups, to determine mutually meaningful priories. These were often supplemented with a literature review or data from performance reports to support the priority areas with a substantial evidence base.

*“Identify priorities that are really meaningful to the teams providing the care with the patients at the centre… [to enhance their motivation to] collect…and use the data to show change of practice.” (Participant* #2, National SCI Registry Director)

Participants stressed that QI priorities need to be tangible and built into current work systems to ensure sustainability and avoid duplication of work.

*[QI strategies] need to be built into replacing things people are currently doing and fit into their work processes.*” (Participant #4, SCI Community Organization Lead)

Participants also suggested the provision of incentives to promote engagement and enhance motivation. Different types of incentives mentioned included: direction from leadership; co-authorship on publications; and financial incentives.

*“We have a publication policy defined in our network… centers that contributed the highest amount of data will be named explicitly in the author list and…that's an incentive [for] people active in the rehabilitation disciplines.” (Participant #10, National SCI Registry Lead)*

#### Interdisciplinary collaboration

All participants discussed interdisciplinary collaboration, including patient and family advisors, as instrumental to successful use and implementation of registry data for QI.

“*There needs to be engagement at a few levels, [including] the program management level, as well as the front line clinician level depending on…[your priority] content area.*” (Participant #4, SCI Community Organization Lead).

The concept of equal partnerships was discussed by a few participants to inspire trust for the QI process and ensure successful collaboration. Specific roles mentioned by participants included health care administrators, leadership, government and policy, front-line workers (e.g., nurses, physiotherapists, occupational therapists, social workers, clinicians, primary care providers, psychologists, recreational therapists, and physiatrists), researchers, QI data experts, and most emphasized, consulting with persons with lived experience (where appropriate and relevant to their experience).

“*It's really important that [the teams] trust the researcher and the researcher makes sure to always be respectful and get their input. It's a two-way street.*” (Participant #5, Federal Government QI Researcher).

#### Champions for accountability and sustainability

Most participants recommended clinical champions to lead QI initiatives. Champions were also seen as useful for identifying appropriate team members and connecting teams to other local, provincial, and national connections.

“*Successful implementation [requires] accountability from leadership to…sustain changes [and] ensure buy in from front line to actually change behaviours.*” (Participant #4, SCI Community Organization Lead)

#### Frequent and on-going communication

Frequent and on-going communication to support QI was emphasized by all participants. Most participants recommended organizing regular meetings in steering committee like structures to facilitate QI. Many participants cautioned that when planning QI to be realistic about the time commitment and consider the busy schedules of clinical professionals.

*“[You should] always be communicating how you’re doing towards your indicators, [and] your best practices. Update the clinicians, [and] bring them together in a way that works for them with their clinical day.*” (Participant #2, National SCI Registry Director)

#### Develop a targeted implementation strategy

Participants recommended tailoring the implementation strategy to the specific research problem, emphasizing on designing discrete tasks.

*“You can’t do it all, it's way too overwhelming…it's really helpful if you can show a team by doing a few things well, [then] they’ll build momentum to add rather than doing all and never starting.*” (Participant #2, National SCI Registry Director)

Participants also suggested identifying available, required resources for implementation including mapping the personnel, time and budget required for impact, to design a feasible implementation strategy. Several participants consulted with individual centers during implementation planning to set expectations, determine site readiness, and contextualize the implementation strategy. Various types of QI strategies were discussed by participants including: external facilitation, educational strategies, and audit and feedback.

“*[We wanted] to understand every centre's…specific issues or concerns or positions individually before we meet them all together so that everybody has a chance to speak their mind.*” (Participant #8, Provincial Research Institute Researcher)

#### Feedback loops and reporting

Feedback loops and reporting were widely discussed by participants as a useful strategy for enhancing QI implementation. Some participants created standard reports across sites to compare across participating sites and against standard or national benchmarks. Some participants recommended collecting data from at least two time points to explore the impact of the QI intervention on decision making or patient outcomes.

“*Those quick feedback loops …are really beneficial and [provide] lots of feedback, so you see how you’re doing, look at what the challenge may be, and then do another cycle.*” (Participant #2, National SCI Registry Director)

Despite the utility of audit and feedback, many participants also noted the resource burden associated with audit and feedback included both time, money, and personnel.

“*The problem with audit and feedback is just that it is expensive… unless you have a person who can sit and make these reports and send them*” (Participant #11, Federal Government Researcher).

There were mixed comments on benchmarking when providing feedback to sites. While some participants felt audit and feedback was a motivator for sites when compared to others, some did not agree and only compared sites with national benchmarks.

“*When you compare to the other centres and then they realize that they did not perform as good. Then they realize that there may be some room for improvement. Comparison is key for constant improvement.*” (Participant #9, Provincial Research Institute)

### Theme 3: operationalization and sustainability

A key enabler to facilitate registry data mobilization for QI was enhancing the operationalization and sustainability of registry data.

#### Determine specific research questions

After identifying mutual areas for improvement, participants suggested determining discrete data sets for key variables that address the research question. It was also recommended to frame data into a larger picture or model of care to highlight the larger impact of the QI initiative, the long-term aspirational goals, and inspire motivation.

*“I think it's looking at key uses for the data and priorities for the data because registries often falter when they try to do too much…” (Participant #3 National SCI Registry Senior Leadership)*

#### Alignment & linking with standards of care

Participants suggested supporting priority research areas with accredited standards of care, best practices, and alignment with international SCI core data measures.

*“[International standards] give a common language for people to talk about, what type of SCI somebody has, how severe it is. It also allows prognostication based on all the literature out there and informs conversations with your patients about outcomes and goal setting.” (Participant #2 National SCI Registry Director)*

#### Long-term sustainability

Participants across jurisdictions described common barriers to implementing QI initiatives that leverage registry data including disconnected health care systems and lack of resources and funding to support QI initiatives.

*“Implementation is a real challenge, because most people have so many good ideas for making health care better, but most of them require time and people to do them.*” (Participant #11, Federal Government Researcher).

Several participants discussed the difficulties of developing and maintaining infrastructure to support QI including the overhead cost and resource burden.

“*It's really hard then to maintain any type of [QI] infrastructure… the maintenance costs are really underestimated.*” (Participant #10, National SCI Registry Lead)

Almost all participants mentioned embedding registry data within electronic medical records (EMR), or linking registry data with other databases, to enhance the utility and sustainability of QI. Some participants discussed registries linked with EMR systems. This process enabled more complete data collection and better characterized complex populations, such as individuals with SCI.

 “*It ultimately has to be sustainable and be embedded into the chart and [electronic health record systems] because otherwise once the money runs out the care stops… You want to make sure it lives on even if the funding ends*.” (Participant #3, National SCI Registry Senior Leadership)

## Synthesis & discussion

This study used a convergent mixed-methods design using a parallel-database variant, combining independent findings and perspectives from a scoping review and qualitative interviews, to better understand the perceived utility, barriers, and facilitators regarding mobilizing registry data for QI amongst interdisciplinary partners. While appreciating that most patient registries are not specifically designed for QI, they can offer robust and diverse data sets to compliment health administrative databases to explore research and QI questions (63–65). The integration of results from a scoping review and qualitative exploration allowed for the identification of three key learnings to enhance the successful design and implementation of QI with interdisciplinary care partners in supporting innovation of rehabilitation care of persons living with complex, chronic conditions: enhance utility and reliability of registry data; form a steering committee lead by clinical champions; and design effective, feasible, and sustainable QI initiatives.

### Enhance utility & reliability of registry data

Many QI initiatives rely on the inherent motivation of providers to participate and engage in change to improve quality of care (66). Findings from both the scoping review and qualitative interviews highlighted that motivating providers to use registry data was a significant barrier to advancing QI initiatives. Common barriers amongst healthcare providers for participating in QI included limited knowledge of how to improve care processes, lack of normative standards and benchmarks, insufficient time to participate and allocate staff, as well as limited capacity to develop and implement appropriate improvement actions (67, 68). Mutual priorities of care, public reporting, and provider incentives were all recommended to enhance provider motivation. Incentives, such as pay-for-performance, to support QI for healthcare has been supported by the Institute of Medicine and others to enhance provider motivation to support QI initiatives (12, 69). Findings from a systematic review found short-term improvements to patient outcomes using pay-for-performance programs but limited benefits for sustainability (70). A 2011 Cochrane review found insufficient evidence to neither recommend for or against financial incentives to enhance primary care quality of care (71). The authors recommended that incentives should be planned and discussed with partners prior to implementation to best support behavior change (71). Incentive programs may be more effective if the desired change is specific, easy to measure, and is focused on interventions with clear room for improvement (72). Other types of incentives, including publications, were also discussed in this review to enhance provider motivation to use and participate in QI without the burden of cost. This may be more feasible in public healthcare systems where access and use of funds is more restricted.

Both the scoping review and qualitative interviews identified mistrust, or perceived unreliability, of registry data as a barrier to support the use of registry data for QI, especially for registry data for complex conditions. Missing information related to specific interventions, outcomes, or prognoses can lead to bias due to non-random selection of data for analysis (73). This was particularly a concern for interview participants when discussing data on individuals with non-traumatic SCI. Significant emphasis from leadership would be required to dedicate the necessary resources needed to support strategies to enhance the reliability and transferability of all registry data, including the addition of data for individuals with non-traumatic SCI. To enhance reliability of the registry, stakeholders from the qualitative interviews recommended to validate the registry data with health system administrative databases to determine whether the patient population described in the registry aligns with those seen at participating care. It was also recommended to link registry data with other databases to include additional metrics to supplement the variables collected by the registry and to compare to standard benchmarks to inform change in policy and practice. Data from a Canadian national SCI registry, the Rick Hansen Spinal Cord Injury Registry (RHSCIR), is captured during the pre-hospital, acute, and rehabilitation phases of cases as well as follow-up in the community at 1, 2, 5, and every 5 years post-injury (30). To support quality improvement of care, RHSCIR has worked with provincial health care partners to link SCI data to multiple databases to address epidemiological and health service research questions as well as better understand patient trajectories (23). This type of registry data linkage can be used to leverage routine administrative or billing databases that are more broadly available diverse health care providers to support continuous data sharing and monitoring (63).

A common barrier of using registry data for QI identified from the scoping review and qualitative interviews was the lack of appropriate information technology supports including incompatibility between registry dashboards and electronic health record systems (EHRs). This represents an ongoing challenge for many health systems, as embedding registry data into EHRs requires significant investment in designing appropriate information technology infrastructure (i.e., hardware, software, personnel etc.) as well as supplying the necessary resources required for monitoring, maintenance, and upgrading over time (26). This has been also noted in primary care, where limitations with EHRs reporting and customizable data reports limited quality measurement reporting and engagement in QI activities (74). In this review, larger national registries or QI groups had either existing registry data collection infrastructures embedded into site EHRs or had sufficient resources to develop and implement these technologies. Embedding registries into EHRs can enable health systems to develop internal registries or contribute valuable health information to external registries including patient reported outcome measures (25). Registry data itself can also further augment available EHR information and allow for comparable safety, effectiveness, efficiency, and other quality reporting measures (25). Registry data infrastructures would need to be streamlined to support quick and easy real-time data entry, be flexible to work at various sites, and run alongside current electronic healthcare record systems or preferably, automatically capture registry data (39, 45, 75). These structures would alleviate the burden of data entry by front-line care providers and enhance registry data useability (45). Additionally, these infrastructures would require dedicated information technology supports to continuously build and support data entry structures over time as well as maintain security and stability of online platforms (45).

### Form a steering committee led by clinical champions

Continuous communication amongst interdisciplinary care partners is essential to ensure effective collaboration. This is especially true for complex conditions, such as SCI, that require collaborative relationships amongst patients; primary care; and interdisciplinary care providers across diverse sectors and services; researchers; policy makers and other key decision makers (10). In this study, successful mobilization of registry data for QI from the scoping review and qualitative interviews commonly involved significant investment from leadership as well as on-going communication and support. The majority of stakeholders interviewed recommended steering committees or other types of working groups to develop, plan, and implement QI. Most initiatives discussed by stakeholders or those identified from the literature recruited sites interested in QI and motivated team leads or champions to spearhead registry data collection and QI processes over time. Despite the recommendation for clinical champions to lead QI initiatives, competing demands for time and lack of previous knowledge in navigating bureaucratic idiosyncrasies of change management within the healthcare system can hinder their success of implementing QI into routine clinical practice. Therefore, when designing QI initiatives clinical champions with previous QI initiatives should be prioritized.

Engaging stakeholders in the design and implementation of QI interventions is essential for mobilizing knowledge to action within the health care system (76, 77). Early and continuous engagement with stakeholders can better support project management, while allowing for the tailoring of QI approaches to support pre-identified barriers and facilitators of implementation (78). To date, there is limited clarity defining stakeholder engagement in implementation science (76). Stakeholders can be involved at any point in the QI approach including the design and specification of the QI purpose, methods, data collection, evaluation, or results interpretation and dissemination (79). In the present study, stakeholders were often consulted during the prioritization and planning stages of the QI intervention. This was observed in both the scoping review and the qualitative interviews. Stakeholders from the qualitative interviews emphasized the need for interdisciplinary stakeholder engagement that included patients at every stage of planning. At the primary care level, registry data can provide up-to-date information on patient chronic illness markers and patient health outcomes (80). It can be used to facilitate data-sharing between providers and patients to better encourage joint decision making, develop metrics reflective of the patient experience, enable the development of individualized patient care plans and goals of care (39, 41). In the scoping review, there was limited mention of patients in the design and evaluation of the QI approach. Traditionally, patient engagement has been restricted to the management of their own care, however, recent efforts are moving towards integrating patients in the re-design and improvement of health service delivery (81–83). To ensure successful implementation and adoption, QI initiatives should focus on continuous engagement with stakeholders, including patient advisors, to ensure their experiences and perspectives are integrated into the design and delivery of health care improvement approaches (83).

### Design effective, feasible, and sustainable QI initiatives

Findings from the scoping review highlighted the diversity of QI approaches used to support change management and evaluation. QI interventions using registry data have been previously described in both the primary care (84, 85) and inpatient rehabilitation setting (27, 30) for persons with complex chronic conditions. Presently, there is unclear evidence to support the design of an effective QI intervention that spans across professional disciplines (86, 87). Due to the variability of study designs, interventions types and outcome measures in the scoping review, it was not feasible to compare or discuss outcomes of individual studies to explore the types of QI approaches that may be more or less effective at using registry data. Most often, the selection of individual QI approaches is justified as beneficial for the study context, design, or based on personal preference or familiarity of the approach (87). Several stakeholders from the qualitative interviews emphasized that successful interdisciplinary QI initiatives were often supported by implementation strategies targeting *a priori* research questions developed from mutual priorities of diverse stakeholder groups that were feasible for the available resources and supports through participating teams.

Findings from the convergent analysis indicated that successful QI strategies were iterative and included multiple touchpoints with teams to enable opportunities for continuous learning and feedback. Audit and feedback was a common method to enhance provider motivation for QI in both the scoping review and the qualitative interviews (88). It can be used to broadly share and clarify quality norms as well as demonstrate the value of QI processes (89). Motivation to use and successfully implement registry data for QI was often spurred by successful and ongoing engagement and data sharing. In this study, audit and feedback was commonly used as a tool to engage sites over time. It provided the QI teams an opportunity to review and improve performance of indicators and evaluate the effectiveness of the QI intervention throughout the study period. One of the reviewed studies described peer assessment as an important audit and feedback strategy that enabled participating sites to learn from each other (60). A caveat of audit and feedback is the significant cost and required personnel to support ongoing data collection and reporting. A recent study found that primary care providers did not perceive audit and feedback as supportive of QI work in their practice (88). Notable barriers to audit and feedback include: criticism of measures used to evaluate quality (including limited measures of clinical quality and patient outcomes); increased demands from growing patient population; and lack of time and resources (88). These findings emphasize the need to develop QI priorities and metrics of success that are meaningful to all stakeholders across diverse disciplines.

### Strengths and limitations

The present convergent analysis aimed to explore approaches to support interdisciplinary collaboration for QI using registry data for complex chronic conditions. This study highlights a diversity of registries and QI activities complimented by the preferences and experiences of international stakeholders to explore the uses, barriers and facilitators, and opportunities to mobilize registry data for QI of rehabilitation care for persons with complex, chronic conditions. The findings of this review are strengthened in that both academic, grey literature, and stakeholder insights were examined to better clarify the uses of registry data for QI. There were few studies or reports exploring the uses of registry for health system QI. SCI specific stakeholders were chosen to support ongoing provincial QI activities. However, inclusion of stakeholders with experience using SCI registries may limit the applicability of these findings to other complex chronic conditions. Future research may require higher level and increased diversity of stakeholder perspectives to understand the nuances of using registry data for QI including stakeholders with expertise in other complex, chronic conditions to investigate the broader transferability of the present findings.

## Conclusion

The learnings from this study provide examples of QI interventions and approaches to developing QI strategies with interdisciplinary care partners to mobilize registry data. These findings suggest the need for interdisciplinary care partners to co-develop appropriate benchmarking and monitoring or outcome evaluation metrics/measures to guide the use of registry data in QI initiatives and other decision making. Enhancing utility and reliability of registry data and forming a steering committee led by clinician champions to design effective, feasible and sustainable QI initiatives through the use of registry data, are strategies that could contribute to overcome known barriers for using data registry in healthcare QI. Understanding the barriers and facilitators in the use of registry data by multidisciplinary partners across care settings can help promote the use of registry data to support the development and implementation of QI initiatives by researchers and clinicians.

## Data Availability

The original contributions presented in the study are included in the article/**Supplementary Material**, further inquiries can be directed to the corresponding author/s.
