# Supplementary material for: Mobilizing registry data for quality improvement: A convergent mixed-methods analysis and application to spinal cord injury
Source: Front Rehabil Sci. 2023 Apr 3;4:899630. doi: 10.3389/fresc.2023.899630 (PMC10109451; doi:10.3389/fresc.2023.899630)
Supplement: Supplementary file 1 [file Table1.docx]

**Appendix A:** Grey Literature Sources

| The National Institute of Health, Agency for Healthcare Research and Quality |
| --- |
| Canadian Institute for Health Information |
| The Canadian Spine Society |
| PRAXIS Spinal Cord Institute |
| New Zealand Spinal Trust |
| Christopher and Dana Reeve Foundation |
| National Spinal Cord Injury Statistical Center |
| Alberta Health Services Insite |
| OAISter |
| IC/ES Data Discovery Better Health |
| Australian Spinal Cord Injury Registry |
| European Multicenter Study About Spinal Cord Injury |
| Quality Enhancement Research initiative |
