# Supplementary material for: Mobilizing registry data for quality improvement: A convergent mixed-methods analysis and application to spinal cord injury
Source: Front Rehabil Sci. 2023 Apr 3;4:899630. doi: 10.3389/fresc.2023.899630 (PMC10109451; doi:10.3389/fresc.2023.899630)
Supplement: Supplementary file 2 [file Table2.docx]

**Appendix B: Search Terms**

| **Concept** | **Synonym** |
| --- | --- |
| Registry Data/Registry Database | “registry data” [Keyword]; “registry database” [Keyword]; “health registry data” [Keyword]; “health registry database” [Keyword]; “clinical registr*” [Keyword]; “clinical registry database” [Keyword]; “patient registr*” [Keyword]; “disease registr*” [Keyword]; “health directory” [Keyword]; “clinical directory” [Keyword]; “patient directory” [Keyword]; “health repositor*” [Keyword]; “clinical repositor*” [Keyword]; “patient repositor*” [Keyword] |
| Registry | registr* [Keyword]; registries [MeSH]; “health registr*” [Keyword]; |
| Data | Data [Keyword]; “data system*” [Keyword]; data systems [MeSH]; public reporting of healthcare data [MeSH]; patient generated health data [MeSH]; “data collection” [Keyword, MeSH]; “data curation” [Keyword, MeSH]; |
| QI | “quality improvement” [Keyword, MeSH]; “quality assurance” [Keyword]; quality assurance, health care [MeSH]; “quality management” [Keyword]; “total quality management” [Keyword, MeSH]; “QIintervention*” [Keyword]; “QIinitiative*” [Keyword]; “QIprogram*” [Keyword]; “organizational innovation” [Keyword, MeSH]; “quality indicator*” [Keyword]; quality indicators, health care [MeSH]; “quality of health care” [Keyword, MeSH]; “continuous improvement” [Keyword]; “continuity of patient care” [Keyword, MeSH]; education [Keyword, MeSH]; “continuing education” [Keyword]; education, continuing [MeSH]; workshop* [Keyword]; audit* [Keyword]; “clinical audit” [MeSH]; “medical audit” [Keyword]; feedback [Keyword, MeSH]; benchmark* [Keyword]; benchmarking [MeSH]; “clinical feedback” [Keyword]; “organizational culture” [Keyword, MeSH]; |
| Spinal Cord Injury (SCI) | SCI [Keyword]; “spinal cord injur*” [Keyword]; spinal cord injuries [MeSH]; tetrapleg* [Keyword]; quadripleg* [Keyword]; quadriplegia [MeSH]; parapleg* [Keyword]; paraplegia [MeSH] |
| Chronic Disease/Complex Chronic Conditions | “chronic disease*” [Keyword]; chronic disease [MeSH]; “complex chronic condition*” [Keyword]; “chronic condition*” [Keyword] |
| Strategy/Approach/Implementation/Outcome | outcome* [Keyword]; “outcome assessment” [Keyword]; outcome assessment, health care [MeSH]; strateg* [Keyword]; approach* [Keyword]; “data accuracy” [Keyword, MeSH]; “data analysis” [Keyword, MeSH]; “data management” [Keyword, MeSH]; “data quality” [Keyword, MeSH]; change [Keyword]; standard* [Keyword]; reference standards [MeSH]; “performance measurement” [Keyword]; evaluat* [Keyword]; evaluation study [MeSH]; “program evaluation” [Keyword, MeSH]; “developmental evaluation” [Keyword, MeSH]; implement* [Keyword]; “health plan implementation” [Keyword, MeSH]; integration [Keyword]; “social integration” [Keyword, MeSH]; “systems integration” [Keyword, MeSH]; “change management” [Keyword, MeSH]; “knowledge translation” [Keyword]; “knowledge transfer” [Keyword]; “information dissemination” [Keyword, MeSH]; “data mobilization” [Keyword] |
