# Supplementary material for: Mobilizing registry data for quality improvement: A convergent mixed-methods analysis and application to spinal cord injury
Source: Front Rehabil Sci. 2023 Apr 3;4:899630. doi: 10.3389/fresc.2023.899630 (PMC10109451; doi:10.3389/fresc.2023.899630)
Supplement: Supplementary file 3 [file Table3.docx]

**Appendix C: Semi-Structured Interview Guide**

1. Where is your primary place of residence?
2. What organization are you currently (or were previously) affiliated with? [*If relevant to participant*]
3. How long have you worked in this role? [*If relevant to participant*]
4. What areas in SCI [*or other disease depending on participant*] care do you think need improvement?
5. What are your thoughts on the types of information collected in SCI patient registries?
6. What types of information is NOT being collected by current registries and what types of information should be collected to better inform the current SCI practices and policies
7. Who should we involve to address this problem?
8. Do you know of any ways health systems can (have) use(d) information from patient registries to address priority issues in SCI care? [*If yes continue to #9, if no, proceed to #9*]
9. Are you aware of any quality improvement strategies developed and implemented based on learnings from registry information? [*If yes continue to #9, if no, proceed to #12*]
10. What types of barriers did you (have you) experience(d) developing or implementing a quality improvement strategy?
11. Based on your experiences, what are (were) the best ways to effectively collaborate within a group to advance quality improvement strategies [or projects in general]?
12. Are there any names of other individuals who could inform this topic?
13. Is there anything more you would like to share on this topic?
