# Supplementary material for: Mobilizing registry data for quality improvement: A convergent mixed-methods analysis and application to spinal cord injury
Source: Front Rehabil Sci. 2023 Apr 3;4:899630. doi: 10.3389/fresc.2023.899630 (PMC10109451; doi:10.3389/fresc.2023.899630)
Supplement: Supplementary file 4 [file Table4.docx]

**Appendix D:** Extracted Articles

| **Author** | **Country of Publication** | **Patient Population** | **Aim(s)** |
| --- | --- | --- | --- |
| Algurén et al., 2019^1^ | Sweden | Heart Failure and Osteoarthritis | To compare teams from two quality improvement collaboratives using national quality registries |
| Asher et al., 2014^2^ | USA | Spine Surgery | To describe the quality reporting outcomes of a national collaborative registry |
| Australian Orthopaedic Association National Joint Replacement Registry AOA PROMs Pilot Project (2020)^3^ | Australia | Joint Replacement | To assess the feasibility of establishing a national data collection platform for patients undergoing joint replacement surgery |
| Burry et al., 2018^4^ | Canada | Type 2 Diabetes | Systematic review of QI strategies that use data from pediatric diabetes registries |
| Caldarella et al., 2012^5^ | Italy | Cancer | To demonstrate the feasibility of evaluating quality of care using cancer registry data |
| Curtis et al., 2020^6^ | New Zealand | Trauma | To establish data use and QI priorities of trauma professionals |
| Dixon-Woods et al., 2020^7^ | USA | Cystic Fibrosis | To explore the views of stakeholders on designing and implementing care informed by registry data |
| Dvorak et al., 2017^8^ | Canada | Spinal Cord Injury | To describe knowledge gaps throughout the spinal cord injury care continuum and propose recommendations to bridge these gaps. |
| Dykes et al., 2015^9^ | USA | Computed Tomography Examination | To develop three national performance benchmarks for intravenous, iodinated, and contrast extravasation during computed tomography examinations |
| Enright et al., 2017^10^ | Canada | Cancer | To develop an approach to prioritize quality measures for improving the quality of systematic therapy for women with early-stage breast cancer |
| Etz et al., 2015^11^ | USA | Type 2 Diabetes | To describe the protocol for the randomized controlled trial: Supporting Practices to Adopt Registry-Based Care (SPARC) |
| Fehlings et al., 2017^12^ | Canada | Spinal Cord Injury | To describe the Access to Care and Timing project, which aims to enhance access and specialized health care delivery for persons with SCI |
| Fonarow et al., 2010^13^ | USA | Heart Failure | To describe the primary results of the registry: To Improve the Use of Evidence-Based Heart Failure Therapies in the Outpatient Setting (IMPROVE HF) |
| Fredriksson et al., 2014^14^ | Sweden | Stroke, Gallstone Surgery, Lung Cancer, | To compare data use for national quality registries in local quality improvement initiatives and explore factors for effective clinical use |
| Fredriksson et al., 2017^15^ | Sweden | Swedish National Quality Registries (General) | To explore the perspectives of politicians and administrators on quality improvement initiatives based on Swedish national registry data |
| Haggstrom et al., 2010^16^ | USA | Cancer | To determine the completeness of registry data for screening, follow-up, and treatment measures and to describe and test practice change in health centers |
| Hoque et al., 2017^17^ | Australia | Clinical Quality Registries (General) | A systematic review exploring the impact of clinical quality registries on morality and survival, processes or outcomes of health care, health care utilization, and health-related costs |
| Kaplan et al., 2018^18^ | USA | Birth | To evaluate the success of a quality improvement initiative to reduce early elective deliveries |
| Lindström Egholm et al., 2019^19^ | Denmark | Cardiac Rehabilitation | To investigate the use of clinical quality data for cardiac rehabilitation for local quality improvement |
| Martin et al., 2019^20^ | USA | Hypoplastic Left Heart Syndrome | To describe the IMproving Pediatric and Adult Congenital Treatments (IMPACT) Registry and the National Pediatric Cardiology Quality Improvement Collective (NPC-QIC) and their potential to improve outcomes for persons with congenital heart conditions. |
| Meerhoff et al., 2017^21^ | Netherlands | Physical Therapy Practice | To describe the development of an implementation strategy to evaluate the feasibility of building registry and implementation patient reported outcome measures in physical therapy practice |
| Noonan et al., 2012^22^ | Canada | Spinal Cord Injury | To describe the framework and methods used to develop the Access to Care and Timing model |
| Prodinger et al., 2018^23^ | England & Sweden | Hip and Knee Joint Replacement | To explore the barriers and facilitators to integrating patient reported outcome measures into health information systems to inform quality of care |
| Sparring et al., 2018^24^ | Sweden | Swedish National Quality Registries (General) | To investigate the perceived barriers and facilitators to using data from national quality registries for quality improvement |
| Thomas et al., 2019^25^ | Australia | Cardiac Rehabilitation | To assess the feasibility of a data capture tool for cardiac rehabilitation to populate a new registry |
| US Department of Veterans Affairs Quality Enhancement Research Initiative (2014)^26^ | USA | Spinal Cord Injury | To provide an overview of the spinal cord injury quality improvement initiatives undertaken by SCI-QUERI |
| White et al., 2017^27^ | USA | Cancer | To overview the history of cancer surveillance programs using registry data |

**Works Cited**

1. Algurén, B., Nordin, A., Andersson-Gäre, B. & Peterson, A. In-depth comparison of two quality improvement collaboratives from different healthcare areas based on registry data - possible factors contributing to sustained improvement in outcomes beyond the project time. *Implement. Sci.* **14**, (2019).

2. Asher, A. L. *et al.* The National Neurosurgery Quality and Outcomes Database (N2QOD). *Spine (Phila. Pa. 1976).* **39**, S106–S116 (2014).

3. Australian Orthopaedic Association National Joint Replacement Registry. *AOA PROMs Pilot Project Final Report*. (2020).

4. Burry, E., Ivers, N., Mahmud, F. H. & Shulman, R. Interventions using pediatric diabetes registry data for quality improvement: A systematic review. *Pediatr. Diabetes* **19**, 1249–1256 (2018).

5. Caldarella, A. *et al.* Feasibility of evaluating quality cancer care using registry data and electronic health records: a population-based study. doi:10.1093/intqhc/mzs020.

6. Curtis, K. *et al.* Priorities for trauma quality improvement and registry use in Australia and New Zealand. *Injury* **51**, 84–90 (2020).

7. Dixon-Woods, M. *et al.* A qualitative study of design stakeholders’ views of developing and implementing a registry-based learning health system. *Implement. Sci.* **15**, 16 (2020).

8. Dvorak, M. F. *et al.* Spinal Cord Injury Clinical Registries: Improving Care across the SCI Care Continuum by Identifying Knowledge Gaps. doi:10.1089/neu.2016.4937.

9. Dykes, T. M., Bhargavan-Chatfield, M. & Dyer, R. B. Intravenous contrast extravasation during CT: A national data registry and practice quality improvement initiative. *J. Am. Coll. Radiol.* **12**, 183–191 (2015).

10. Enright, K. A. *et al.* Setting quality improvement priorities for women receiving systemic therapy for early-stage breast cancer by using population-level administrative data. *J. Clin. Oncol.* **35**, 3207–3214 (2017).

11. Etz, R. S. *et al.* Supporting practices to adopt registry-based care (SPARC): Protocol for a randomized controlled trial. *Implement. Sci.* **10**, 46 (2015).

12. Fehlings, M. G. *et al.* Using Evidence To Inform Practice and Policy To Enhance the Quality of Care for Persons with Traumatic Spinal Cord Injury. *J. Neurotrauma* **34**, 2934–2940 (2017).

13. Fonarow, G. C. *et al.* Improving evidence-based care for heart failure in outpatient cardiology practices: Primary results of the registry to improve the use of evidence-based heart failure therapies in the outpatient setting (IMPROVE HF). *Circulation* **122**, 585–596 (2010).

14. Eldh, A. C. *et al.* Facilitators and barriers to applying a national quality registry for quality improvement in stroke care. *BMC Health Serv. Res.* **14**, 1–10 (2014).

15. Fredriksson, M. *et al.* Are data from national quality registries used in quality improvement at Swedish hospital clinics? *Int. J. Qual. Heal. Care* **29**, 909–915 (2017).

16. Haggstrom, D. A., Clauser, S. B. & Taplin, S. H. The health disparities cancer collaborative: A case study of practice registry measurement in a quality improvement collaborative. *Implement. Sci.* **5**, 42 (2010).

17. Md, D. *et al.* Impact of clinical registries on quality of patient care and clinical outcomes: A systematic review. (2017) doi:10.1371/journal.pone.0183667.

18. Kaplan, H. C. *et al.* Statewide Quality Improvement Initiative to Reduce Early Elective Deliveries and Improve Birth Registry Accuracy. *Obstet. Gynecol.* **131**, 688–695 (2018).

19. Egholm, L. Facilitators for using data from a quality registry in local quality improvement work: a cross-sectional survey of the Danish Cardiac Rehabilitation Database. *BMJ Open* **9**, 28291 (2019).

20. Martin, G. R., Anderson, J. B. & Vincent, R. N. IMPACT Registry and National Pediatric Cardiology Quality Improvement Collaborative: Contributions to Quality in Congenital Heart Disease. *World J. Pediatr. Congenit. Heart Surg.* **10**, 72–80 (2019).

21. Meerhoff, G. A. *et al.* Development and evaluation of an implementation strategy for collecting data in a national registry and the use of patient-reported outcome measures in physical therapist practices: Quality improvement study. *Phys. Ther.* **97**, 837–851 (2017).

22. Noonan, V. K. *et al.* The Application of Operations Research Methodologies to the Delivery of Care Model for Traumatic Spinal Cord Injury: The Access to Care and Timing Project. doi:10.1089/neu.2012.2317.

23. Prodinger, B. & Taylor, P. Improving quality of care through patient-reported outcome measures (PROMs): expert interviews using the NHS PROMs Programme and the Swedish quality registers for knee and hip arthroplasty as examples. doi:10.1186/s12913-018-2898-z.

24. Sparring, V., Granström, E., Andreen Sachs, M., Brommels, M. & Nyström, M. E. One size fits none-a qualitative study investigating nine national quality registries’ conditions for use in quality improvement, research and interaction with patients. doi:10.1186/s12913-018-3621-9.

25. Thomas, E. *et al.* Utilising a Data Capture Tool to Populate a Cardiac Rehabilitation Registry: A Feasibility Study. *Hear. Lung Circ.* **29**, 224–232 (2020).

26. VHA Office of Research and Development. *Quality Enhancement Research Initiative (QUERI) Strategic Plan*. www.hsrd.research.va.gov/queri (2015).

27. White, M. C. *et al.* The history and use of cancer registry data by public health cancer control programs in the United States. *Cancer* **123**, 4969–4976 (2017).
