# Supplementary material for: Mobilizing registry data for quality improvement: A convergent mixed-methods analysis and application to spinal cord injury
Source: Front Rehabil Sci. 2023 Apr 3;4:899630. doi: 10.3389/fresc.2023.899630 (PMC10109451; doi:10.3389/fresc.2023.899630)
Supplement: Supplementary file 5 [file Table5.docx]

**Appendix E: Underlying Theoretical or Methodological Quality Improvement (QI) Frameworks**

| **QI Initiative** | **Definition** |
| --- | --- |
| Plan-Do-Study-Act (PDSA) Cycles | An iterative QI methodology to facilitate change. ‘Plan’ning consists of developing the test or implementation for change. This stage includes specifying the objective, identifying expected outcomes, and planning what is needed to test the implementation (e.g., who, what where, when?). ‘Do’ involves implementing or testing the study. ‘Study’ includes analyzing and evaluating the study results and formulating lessons learned. Understanding the results of the test enables organizations to ‘Act’ by refining or changing the test protocols to improve the outcomes and implementation process.^1^ PDSA cycles are either used as a standalone method or used as part of a wider QI approach.^2^ |
| Continuous Quality Improvement | An incremental process that promotes improvement of processes, safety, and patient care. This process commonly includes defining areas of improvement, identifying benchmarks of success, iterative QI projects, and evaluation of success. PDSA cycles are often used in continuous QI initiatives.^3^ |
| The Breakthrough Collaborative Model | A short-term (6-15 month) initiative designed by the Institute of Health Improvement (IHI) that brings together multidisciplinary teams to make ‘breakthrough’ improvements in a specific area. Collaboratives consist of several teams (12-160 teams). Each team selects members to attend learning sessions (face-to-face meetings), and work on improvements for their local organization. Key elements of the model include pre-work activities (e.g., topic selection, determining team membership), learning sessions (e.g., coaching/mentoring, develop action plans to overcome identified barriers), action periods (test and implement approaches to change and collect data to measure the impact of change), models for improvement, and measurement and evalution.^4–6^ |
| Grol and Wensing Implementation of Change Model | An implementation process model based on a synthesis of theories concerning behaviour change. This model describes theories relevant to describing and explaining effective implementation at the level of the individual (e.g., cognitive, education), social context (e.g., social learning, leadership, patient influence), and organizational and economic context (e.g., organizational learning, quality management, reimbursement).^7^ |
| Knowledge to Action Process | A conceptual framework consisting of two components with multiple iterative phases: knowledge creation and an action cycle. The action cycle describes the process and activities needed to facilitate knowledge integration into practice. It involves adapting knowledge to the local context; collaborating with stakeholders; identifying barriers and facilitators to implementation; and tailoring knowledge to the needs of end-users. Action cycles are often impacted by knowledge creation and can be carried out sequentially or simultaneously.^8,9^ |
| Practice Quality Improvement | A process whereby clinical practitioners, their practices, or their facilities action and document the quality of care provided to their patients to identify priority areas of care and developed targeted improvement strategies.^10^ |
| QI Collaborative | A multifaceted approach that focuses on improving clinical outcomes. QI collaboratives are used in various clinical areas, and organizational contexts and combine clinical and QI experts to support and facilitate implementation. These collaboratives often includes structured activities including: defining the problem; creating a model for improvement; and a series of structured activities to address or improve the problem.^4,11^ |

**Works Cited:**

1. Institute for Healthcare Improvement. Science of Improvement: Testing Changes. http://www.ihi.org/resources/Pages/HowtoImprove/ScienceofImprovementTestingChanges.aspx (2020).

2. Taylor, M. J. *et al.* Systematic review of the application of the plan-do-study-act method to improve quality in healthcare. doi:10.1136/bmjqs.

3. O’Donnell, B. & Gupta, V. *Continuous Quality Improvement*. *StatPearls* (StatPearls Publishing, 2021).

4. T Schouten, L. M. *et al.* Evidence for the impact of quality improvement collaboratives: systematic review. doi:10.1136/bmj.39570.749884.BE.

5. The Breakthrough Series: IHI’s Collaborative Model for Achieving Breakthrough Improvement. *Diabetes Spectr.* **17**, 97–101 (2004).

6. Beers, L. S. *et al.* Mental health screening quality improvement learning collaborative in pediatric primary care. *Pediatrics* **140**, (2017).

7. Grol, R., Wensing, M., Eccles, M. & Davis, D. Improving patient care; the implementation of change in health care. *Second Edition ed. Oxford: Wiley Blackwell* vol. 2nd;Second (2013).

8. Graham, I. D. *et al.* Lost in knowledge translation: time for a map? *J. Contin. Educ. Health Prof.* **26**, 13–24 (2006).

9. Field, B., Booth, A., Ilott, I. & Gerrish, K. Using the Knowledge to Action Framework in practice: a citation analysis and systematic review. *Implementation science : IS* vol. 9 172 (2014).

10. Dykes, T. M., Bhargavan-Chatfield, M. & Dyer, R. B. Intravenous contrast extravasation during CT: A national data registry and practice quality improvement initiative. *J. Am. Coll. Radiol.* **12**, 183–191 (2015).

11. Algurén, B., Nordin, A., Andersson-Gäre, B. & Peterson, A. In-depth comparison of two quality improvement collaboratives from different healthcare areas based on registry data - possible factors contributing to sustained improvement in outcomes beyond the project time. *Implement. Sci.* **14**, (2019).
