# Supplementary material for: Mobilizing registry data for quality improvement: A convergent mixed-methods analysis and application to spinal cord injury
Source: Front Rehabil Sci. 2023 Apr 3;4:899630. doi: 10.3389/fresc.2023.899630 (PMC10109451; doi:10.3389/fresc.2023.899630)
Supplement: Supplementary file 6 [file Table6.docx]

**Appendix F: Exemplar Quotes**

| **Registry Data Access and Validity** | |
| --- | --- |
| **Limited High Quality SCI Evidence** | “The barrier was that there wasn’t that many things that had high-level evidence that we felt, at least in SCI, we should be pushing for implementation” (Participant #5, Federal Government QI Researcher)  “Everyone was only interested in [data on] traumatic [SCI] patients, which I think is a pity because it’s not the majority of our patients anymore.” (Participant #10, National SCI Registry Lead) |
| **Registry Data Access, and Reliability** | “I honestly think [with] registry data it’s 80% working with the data and data management and [20% for] the … [data] analysis.”(Participant #3, National SCI Registry Senior Leadership) |
| **Data Validation** | “If you don’t validate with the center and the people that are practicing you won’t be able to make sure your data is okay.” (Participant #9, Provincial Research Institute Researcher)  “We spent a lot of time on data cleaning, validating, and data management in terms of understanding the databases and back and forth with the data stewards to make sure we knew what we were looking at.” (Participant #3, National SCI Registry Senior Leadership) |
| **Communication and Collaboration** | |
| **Provider Motivation and Competing Priorities** | “We are trying to engage the front-line staff… so they have that built in feedback about what they are doing as opposed to [using the] registry as a research study trying to dictate practice.” (Participant #5, Federal Government QI Researcher)  “It’s hard to get anyone to see the value of [the data], but [seeing it in] the context of a bigger picture changed their care…they start to get excited of the role that the data is playing.” (Participant #3, National SCI Registry Senior Leadership) |
| **Mutual Priorities and Incentives** | “All those care providers [need] to understand why they’re doing this…You bring them along in all of those points along the way rather than push something on them to collect, because I don’t think that is going to create the value.” (Participant #3, National SCI Registry Senior Leadership)  “Some of it is based on the literature, and some of it is based on input from stakeholders, clinicians, patients, who identify issues that they are having ...” (Participant #5, Federal Government QI Researcher) |
| **Interdisciplinary Collaboration** | “You need the engagement from front line, the administrative buy in, and then it needs to be built into replacing things people are currently doing, fit into their work processes, and all of that to be able to actually be sustained.” (Participant #5, Federal Government QI Researcher)  “Partnering with community stakeholders [is] huge because we are all working for the same goal and we can all share the data… [and] come up with creative solutions together without duplication.” (Participant #1, SCI Community Organization Lead) |
| **Champions for Accountability & Sustainability** | “We would get clinical champions at the sites together to problem solve, share solutions, [and] identify problems that they were encountering” (Participant #5, Federal Government QI Researcher)  “It’s really important to have somebody at the [participating] facilities…whose role is to monitor and lead change.” (Participant #2, National SCI Registry Director) |
| **Frequent and On-Going Communication** | “It’s a two way street [where] both people don’t hesitate to reach out to the other and offer support.” (Participant #1, SCI Community Organization Employee)  “Sharing parts of your story along the way can find those [individuals] that can help you with any challenges you are having or there may be people who want to follow your lead. It might be just a venue beyond the typical publishing.” (Participant #4, National SCI Registry Director) |
| **Develop a Targeted Implementation Strategy** | “First, [we] met with every center [individually] to understand their own specific issues or concerns … so we had broad idea of what’s important and what’s less important for them…After [that], we had one meeting with the whole consulting committee [and] presented the variables that we wanted to evaluate and made them discuss if there are some things that we should add or discard.” (Participant #9, Provincial Research Institute Researcher)  “Once you get your goals for what the focus will be, then you can work backwards from there of what your activities will be.” (Participant #2 National SCI Registry Senior Leadership) |
| **Feedback Loops and Reporting** | “Every center got their own data and we would discuss the data with them and at meetings [where] they’ll see [data] from other centers.” (Participant #8, Provincial Research Institute Researcher)  “They had put together this list of performance metrics primarily around preventative care that they wanted to monitor…the centers were very aware that they were being monitored…[and] I think they were actually kind of relieved that we were going to help them address these gaps [in care].” (Participant #5, Federal Government QI Researcher) |
| **Operationalization and Sustainability** | |
| **Determine Specific Research Questions** | “I think that registry data [has] very, very interesting data, but there needs to be some hypothesis behind it. I think that’s important because otherwise you’re documenting a lot of things which are then late and not really used.” (Participant #10, National SCI Registry Lead)  “[Define] a common question that everyone want[s] to answer, [but] nobody has the amount of data needed to answer that [question] on an individual facility level.” (Participant #4, SCI Community Organization Lead) |
| **Alignment & Linking with Standards of Care** | “As a program, it’s really important to identify what are the priorities, and if they are established nationally…so they can get some success with implementation” (Participant #3, National SCI Registry Senior Leadership)  “We wanted to leverage regional and national initiatives. If there [is] an effort nationally going on for something [you’re working on], maybe [you] should back that up.” (Participant #11, Federal Government Researcher)  “You have a gold standard for [your priority areas] and you [can monitor] each center to make sure that they collect the data and they get interested in their data.” (Participant #9, Provincial Research Institute Researcher) |
| **Long-Term Sustainability** | “It is a real challenge because most people have so many good ideas for making health care better but most of them require time and people to do them.” (Participant #11, Federal Government Researcher)  “In terms of sustainability [it] is a really hard goal…you need a minimum amount of money in order to maintain your IT infrastructure and [you need] to have a person that is taking care of the support centers [and] quality reports.” (Participant #10, National SCI Registry Lead)  “[We wanted to] develop a registry that pulls information from the electronic medical record and then supplements that [with] information that is not routinely collected in a systematic way” (Participant #5, Federal Government QI Researcher) |
